# Development of a Loop-Mediated Isothermal Amplification (LAMP) Assay Targeting the Citrate Synthase Gene for Detection of *Ehrlichia canis* in Dogs

**DOI:** 10.3390/vetsci7040156

**Published:** 2020-10-15

**Authors:** Angela Patricia B. Chua, Remil L. Galay, Tetsuya Tanaka, Wataru Yamazaki

**Affiliations:** 1 Department of Veterinary Paraclinical Sciences, College of Veterinary Medicine, University of the Philippines Los Baños, College, Laguna, Los Baños 4031, Philippines; abchua5@up.edu.ph; 2 Laboratory of Infectious Diseases, Joint Faculty of Veterinary Medicine, Kagoshima University, Korimoto 1-21-24, Kagoshima 890-0065, Japan; 3 Center for Southeast Asian Studies, Kyoto University, Shimoadachi-cho 46, Yoshida, Sakyo-ku, Kyoto 606-8501, Japan; yamazaki@cseas.kyoto-u.ac.jp

**Keywords:** *Ehrlichia canis*, canine monocytic ehrlichiosis, citrate synthase gene, loop-mediated isothermal amplification

## Abstract

Canine monocytic ehrlichiosis caused by *Ehrlichia canis* is one of the leading tick-borne diseases of dogs, particularly in tropical countries. A highly sensitive and specific diagnostic method is essential for early detection to facilitate treatment. This study was conducted to develop *E. canis* loop-mediated isothermal amplification (LAMP) assay, a highly sensitive yet simple molecular technique, targeting the citrate synthase (*gltA*) gene of *E. canis*. Canine blood samples were subjected to conventional PCR targeting *E. canis gltA*. After analysis of the sequences of PCR amplicons, LAMP primers were generated. The optimum temperature and time for the LAMP assay were determined using eight samples—after which, the effectiveness and reproducibility of LAMP were verified by testing 40 samples, which included PCR-positive and negative samples. The detection limit was also established. The optimal condition for the assay was 61 °C for 60 min. Compared to PCR, the LAMP assay had a relative sensitivity and specificity of 92.5 and 100%, respectively. Statistical analysis using McNemar’s test showed that the *E. canis* LAMP assay has no significant difference with PCR. Therefore, the LAMP assay developed in this study may be used as an alternative to PCR in the detection of *E. canis*.

## 1. Introduction

Canine monocytic ehrlichiosis (CME) is caused by *Ehrlichia canis*, an obligate intracellular tick-borne pathogen of dogs primarily transmitted by the brown dog tick *Rhipicephalus sanguineus* [1,2]. CME is a multisystemic disease usually characterized by thrombocytopenia, which leads to hemorrhagic disorders, as well as lymphadenomegaly, splenomegaly, and ophthalmologic disorders [3]. In severe cases, bleeding tendencies, including petechiae and ecchymoses with pancytopenia, may occur and eventually lead to death [4]. The occurrence of CME in Southeast Asia has been long established [5]. Recent studies utilizing PCR showed that *E. canis* is a leading tick-borne pathogen of dogs in Southeast Asia [6,7,8,9] and has been associated with severe anemia [10,11].

Early diagnosis is crucial in prompt control of the disease since it can progress to a mild or severe chronic phase with a grave prognosis [12]. The clinical signs of CME are similar to that of other tick-borne diseases; hence, diagnostic tools that can specifically demonstrate the pathogen are necessary. Detection of *E. canis* is conventionally done by microscopic examination of blood smear. However, it is difficult to diagnose it in the acute phase of the infection due to the low number of morulae [3]. PCR is a highly sensitive molecular technique capable of detecting *E. canis* DNA as early as 4–10 days post-infection [13]. However, PCR is mostly applied in research since it is time-consuming, sophisticated, and labor-intensive [14]. In addition, it has limited use in clinical settings due to the need for expensive equipment and adequate skills.

The loop-mediated isothermal DNA amplification (LAMP) is also a highly sensitive molecular technique but is more rapid and more uncomplicated than PCR. In LAMP, a target gene is amplified using four to six primers under an isothermal condition within an hour or less [15]. LAMP reaction may be interpreted visually based on turbidity or fluorescence upon addition of intercalating dyes. LAMP products can also be subjected to gel electrophoresis to examine for the presence of multiple bands with a ladder-like appearance [16]. A colori-fluorometric indicator (CFI) developed by Hayashida et al. allows straightforward interpretation based on the visualization of a color change under white light or observation of fluorescence with the aid of ultraviolet light [17]. Some studies reported LAMP development for the detection of *E. canis* that targeted the heat shock operon *groESL* gene [18] and *p30* gene [14,19]. The use of LAMP in the detection of *E. canis* in the Philippines has not been investigated. In this study, the development of a LAMP assay targeting the citrate synthase gene (*gltA*) for detecting *E. canis* in canine blood was investigated. The detection method mentioned may be possibly adapted in veterinary clinics in the Philippines and other countries.

## 2. Materials and Methods

### 2.1. Blood Samples and DNA Extraction

The canine blood samples used in this study have been previously collected from veterinary clinics in cities in Metropolitan Manila, the capital region of the Philippines, and tested using conventional PCR in a previous study [9]. A total of 46 *E. canis*-positive and 12 *E. canis*-negative samples based on PCR were identified. DNA was extracted from the blood samples using a commercial spin-column based extraction kit (GeneALL^®^ Exgene^TM^ Blood SV mini GeneALL Biotechnology Co., Ltd., Seoul, Korea) following the manufacturer’s protocol. To confirm successful DNA extraction, amplification of the control *actin* gene was carried out as described previously [9] using the Tks Gflex DNA Polymerase (Takara, Kusatsu, Shiga, Japan).

### 2.2. Conventional PCR for E. canis and Sequence Analysis

DNA samples were subjected to a conventional PCR targeting the citrate synthase or *gltA* gene of *E. canis* following the conditions described by Inokuma et al. [20]. Table 1 depicts the primers utilized. The PCR products were separated by electrophoresis on 2% agarose gel in 1 x TAE buffer, followed by staining with ethidium bromide. Bands with a size of approximately 1251 bp were considered positive. Using Nucleospin^®^ Gel and PCR clean up kit (Macherey-Nagel, Dueren, Germany), twelve positive amplicons were subjected to sequencing following purification according to the manufacturer’s protocol.

The sequences of the selected *E. canis gltA* amplicons were compared to that of reported isolates using the Standard Nucleotide Basic Local Alignment Search Tool (BLAST) program (http://blast.ncbi.nlm.nih.gov). Multiple alignments were accomplished to determine similarity among amplicons.

### 2.3. LAMP Primer Design and Optimization of LAMP Condition

The consensus sequence of *E. canis gltA* from selected samples was used to generate LAMP primers using the online LAMP primer designing software, Primer Explorer version 5 (http://primerexplorer.jp/e/) (Table 1).

LAMP reaction mixtures were prepared by mixing 2.5 µL (1×) 10× isothermal amplification buffer, 6 mM MgSO_4_, 1.4 mM dNTP mix, a primer mix containing 0.2 µM F3/B3 primers, 1.6 µM FIP/BIP primers, and 0.4 µM LF/LB primers, 8 U *Bst* 2.0 WarmStart^®^ DNA Polymerase (New England Biolabs, Inc., Ipswich, MA, USA), 1 µL colori-fluorometric indicator (CFI), 2 µL DNA, and nuclease-free water to achieve a final volume of 25 µL. CFI contains 3 mM hydroxylnaphthol blue (HNB; MP Biomedicals, Aurora, OH, USA) and 0.35% *v*/*v* GelGreen (10 000 × Sol, Biotium, Hayward, CA, USA) dissolved in distilled water [17]. For optimizing LAMP condition, six PCR-positive and two PCR-negative samples were subjected to a LAMP assay for 60 min with varying temperatures between 60 °C to 65 °C. A negative control (nuclease-free water) was included in each run. The reaction was then terminated by heating at 80 °C for two minutes. After determining the optimum temperature, reaction time was varied to 30, 45, and 60 min.

### 2.4. LAMP Reproducibility and Detection Limit

The remaining 40 *E. canis* PCR-positive samples and ten negative samples underwent LAMP assay using the optimized temperature and time. Analysis of LAMP products through gel electrophoresis was also performed similarly to what was described earlier after PCR. To determine the detection limit, a purified PCR amplicon of *E. canis gltA* (7.41 × 10^9^ copies) was serially diluted 10-fold up to 7.41 × 10^4^ copies being the lowest concentration, and was subjected to LAMP.

### 2.5. Data Analysis

To compare LAMP and PCR, Mc Nemar’s test was applied using a 2 × 2 contingency table. The relative sensitivity, specificity, accuracy, and positive and negative predictive values were calculated, of which all were expressed as a percentage. The significant difference between LAMP and conventional PCR was set at *p* < 0.05.

## 3. Results

### 3.1. PCR and Sequence Analysis

The targeted 1251 bp fragment of *E. canis gltA* was successfully amplified through conventional PCR. BLAST analysis showed that the selected amplicons shared 93–99% identity with *E. canis gltA* sequences stored in GenBank. Meanwhile, multiple alignments revealed a 96% sequence similarity among the amplicons. The LAMP primers were designed based on the consensus sequence of the PCR amplicons.

### 3.2. Optimum LAMP Temperature and Time

**Figure 1:**
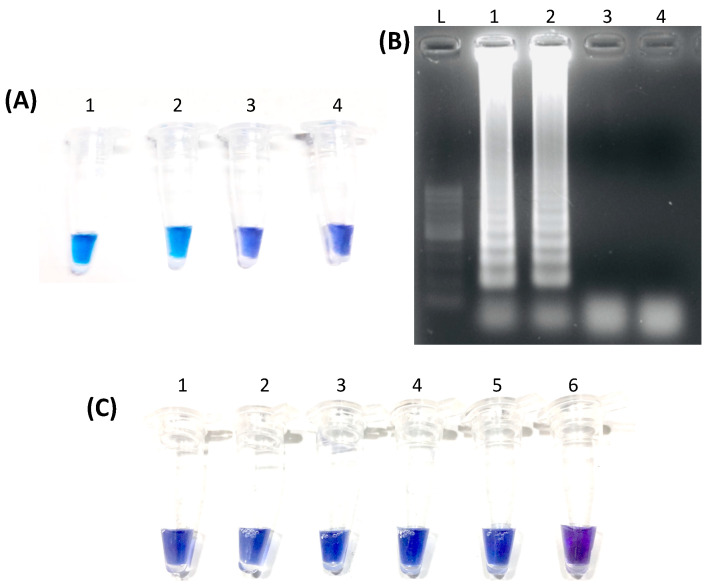
Representative results of LAMP assay targeting *E. canis gltA* by direct visualization and after gel electrophoresis. (**A**) shows the results of selected samples under the established optimal condition through direct visualization wherein Tubes 1 and 2 show a blue color indicative of a positive reaction. In contrast, Tube 3 shows a violet color indicative of a negative reaction similar to the negative control (Tube 4). In (**B**), the same samples were subjected to gel electrophoresis and viewed over a UV transilluminator. Lanes 1 and 2 display multiple bands with a ladder-like appearance, similar to the DNA marker (L), whereas Lane 3 shows the absence of bands, similar to the negative control in Lane 4. (**C**) shows the test results for the detection limit using a purified PCR amplicon of *E. canis gltA* that was serially diluted 10-fold. Tube 1 has the highest number of template copies at 7.41 × 10^9^ while tube 5 has the lowest with 7.41 × 10^4^ copies. Tube 6 is the negative control.

The results of LAMP in different temperatures were compared to that of the PCR (Table 2). LAMP at 60 °C, 61 °C, and 64 °C matched the PCR results of all tested samples. However, among those temperatures, an apparent distinction between the positive and negative reactions indicated by a blue and violet color, respectively, was most evident at 61 °C (Figure 1A). Visualization after gel electrophoresis of selected positive samples showed a ladder-like appearance while the negative sample and negative control showed no bands (Figure 1B). After establishing the optimum temperature, the duration of the LAMP reaction was varied. At various incubation times, all samples matched the PCR results with 60 min having the most evident color distinction between positive and negative results. Hence, the optimal condition for LAMP was set at 61 °C for 60 min.

### 3.3. LAMP Reproducibility and Detection Limit

The reproducibility and effectiveness of developed *E. canis* LAMP were tested by running 40 PCR positive and ten negative samples. Among the PCR-positive samples, 37 tested positive using LAMP, while three tested negative (Table 3). Meanwhile, all PCR negative samples were also negative in LAMP. Regarding the detection limit, a purified *gltA* fragment amplicon was serially diluted ten-fold, with the number of copies ranging from 7.41 × 10^9^ being the highest and 7.41 × 10^4^ being the lowest. All concentrations tested positive in LAMP (Figure 1C).

### 3.4. LAMP Sensitivity, Specificity, and Accuracy

Based on the obtained results, LAMP has 92.5% relative sensitivity, 100% relative specificity, and 94% accuracy compared to conventional PCR. Its positive and negative predictive values are 100 and 76.92%, respectively. Mc Nemar’s test showed no significant difference (*p* = 0.12411) between the two methods.

## 4. Discussion

CME due to *E. canis* is widespread, most likely due to its vector tick’s worldwide distribution [21]. Our recent study suggested that CME is the leading TBD of dogs in the Philippines [7]. Aside from causing significant morbidities and fatalities in dogs, *E. canis* is recognized as a potential zoonotic pathogen after being reported in some cases in humans [22,23,24]. Early diagnosis is crucial to control the progress of infection promptly. Being superior to microscopic examination in terms of sensitivity and a better indicator of active disease than antibody detection, PCR is useful in early detection of *E. canis* infection [25]. However, the time requirement and sophistication hinder its use in the veterinary clinic setting as a routine diagnostic tool, particularly in developing countries like the Philippines. Thus, the development of a LAMP assay for *E. canis* detection due to its simplicity, shorter time requirement, and lower cost compared to PCR was further explored.

In this study, LAMP primers were designed based on an amplified fragment of the *E. canis gltA* gene using conventional PCR. Previous studies that developed LAMP assay for *E. canis* targeted *groESL* [18] and *p30* [14,19]. *gltA* is an ideal target because of the lower level of similarity among *Ehrlichia* species and related genera compared to *groESL* and *16sr RNA* genes [20,26]. Therefore, the use of this gene is beneficial in the detection of specific species.

The use of CFI with HNB as a dye enabled the immediate and easy visual interpretation of LAMP results in this study without requiring any equipment. In the case of a positive reaction, the color change from violet to blue occurs due to the decrease in Mg^2+^ ion concentration upon chelation by dNTPs [27]. The presence of multiple bands with a ladder-like appearance after gel electrophoresis of positive samples supports the visual interpretation. In this study, several temperatures and reaction times yielded similar results. However, the optimal reaction condition, 61 °C for 60 min, was decided based on the distinct difference in the color of the positive samples from the negative samples. Using LAMP, the total testing time from DNA extraction to the visualization of results was accomplished in only two hours and 20 min compared to that of PCR, which took more than four hours. Although a thermal cycler was used to carry-out the LAMP reactions, a heat block may be used as a substitute to simplify the equipment requirement. Moreover, with the use of CFI, lysed blood can be utilized as starting material without DNA extraction [17] to simplify further and shorten the process.

Among the 40 PCR positive samples, three samples were negative in LAMP. The false-negative result may be due to the sequence variation in the DNA target. Since more primers were used in a LAMP reaction, accurate hybridization at nucleotide to nucleotide bases is needed to facilitate strand displacement DNA amplification [19]. The LAMP primers were designed based on the consensus sequence of twelve amplicons. Those three samples may have some differences in the nucleotide sequence compared to that of the other samples. The sequence variation may be due to differences among strains of *E. canis* [28,29]. Another possible reason proposed by Pinhanelli et al. [14] could be the inhibitory effect of the DNA components of the samples.

Compared with conventional *E. canis* PCR, the LAMP reaction in this study had a 92.5% relative sensitivity and 100% relative specificity. It also has a 94% (47/50) accuracy. These values were higher than those obtained in the study of Pinhanelli et al. [14] and comparable to that of Muangchuen et al. [19], which used LAMP targeting *p30* gene. Mc Nemar’s test revealed that the difference between conventional PCR and LAMP was statistically insignificant. This implies that the LAMP is comparable with PCR in the detection of *E. canis*, and thus the LAMP assay targeting *gltA* can be used as an alternative to PCR for the detection of *E. canis*. In studies on the detection of other members of Anaplasmataceae, the LAMP assay was also found to have displayed superior performance over PCR [30,31,32].

To summarize, a LAMP assay targeting the *gltA* gene was developed for the detection of *E. canis*. The LAMP primers, designed based on the sequences of selected PCR positive amplicons, were proven to be effective, with an optimum reaction condition at 61 °C for 60 min. The use of CFI allowed a rapid and straightforward interpretation of results. Based on the statistical analysis, there was no significant difference between the two molecular techniques, making LAMP a comparable diagnostic tool to conventional PCR. Therefore, this assay may be done as an alternative to PCR in the detection of *E. canis* and can be adapted for use in veterinary clinics to aid in the accurate diagnosis of CME. Future investigation should be aimed at increasing the sensitivity of this assay to avoid having false-negative results. The sequences of PCR-positive samples that turned negative in LAMP should be examined for variations, and the primer sequences should be redesigned accordingly to ensure the detection of multiple strains of *E. canis*. The testing of a large number of clinical samples is recommended to assess its effectiveness. Further comparative study of LAMP and PCR may look into the earliest time the pathogen is detectable during the disease onset. A heat block or a regular laboratory water bath can be substituted for a thermal cycler to simplify the material requirement. Lastly, the use of lysed blood samples as starting material may be considered to shorten the time required.

## Figures and Tables

**Table 1:** List of primers targeting *Ehrlichia canis gltA* used in PCR and LAMP.

Method	Primer Name	Sequence (5′ → 3′)	Reference
PCR	Ecanis Fw	TTATCTGTTTGTGTTATATAAGC	[20]
	Ecanis Rev	CAGTACCTATGCATATCAATCC	
LAMP	Ecanis F3	GCTGATCATGAGCAAAATGC	This study
	Ecanis B3	GCCTCGTACTTTTATTACCATCT	
	Ecanis FIP	TCCCTGCTACCAAACAAGCAAGCTACTGTTAGGTTGGCTG	
	Ecanis BIP	AGCTCATGGTGGTGCTAATGAATTGAATAAACTGCTTTACGTTAC	
	Ecanis LF	ATAAGTCAGCACCAGAAGAAC	
	Ecanis LB	GCTGTGATTAATATGTTAATGGCA	

**Table 2:** Result of LAMP assay for *E. canis* using different temperatures.

Sample ID		PCR Result	LAMP Temperature (°C)					
			60	61	62	63	64	65
1	BESC5	+	+	+	+	+	+	−
2	BBV3	+	+	+	+	+	+	−
3	PSY20	+	+	+	+	+	+	−
4	D8	+	+	+	+	+	+	−
5	D25	+	+	+	+	+	+	+
6	D26	+	+	+	+	+	+	−
7	BHK2	−	−	−	−	−	−	−
8	D45	−	−	−	+	+	−	−
9	Neg Control	−	−	−	−	−	−	−

**Table 3:** Contingency table for the comparison of LAMP and PCR in the detection of *E. canis*.

	PCR Positive	PCR Negative	Total
LAMP positive	37	0	37
LAMP negative	3	10	13
Total	40	10	50
